# Predictors for reactogenicity and humoral immunity to SARS-CoV-2 following infection and mRNA vaccination: A regularized, mixed-effects modelling approach

**DOI:** 10.3389/fimmu.2023.971277

**Published:** 2023-02-09

**Authors:** Erin C. Williams, Alexander Kizhner, Valerie S. Stark, Aria Nawab, Daniel D. Muniz, Felipe Echeverri Tribin, Juan Manuel Carreño, Dominika Bielak, Gagandeep Singh, Michael E. Hoffer, Florian Krammer, Suresh Pallikkuth, Savita Pahwa

**Affiliations:** ^1^ Department of Otolaryngology, University of Miami Miller School of Medicine, Miami, FL, United States; ^2^ Department of Microbiology and Immunology, University of Miami Miller School of Medicine, Miami, FL, United States; ^3^ Department of Biomedical Engineering, University of Miami, Miami, FL, United States; ^4^ Department of Microbiology, Icahn School of Medicine at Mount Sinai, New York, NY, United States; ^5^ Department of Neurological Surgery, University of Miami, Miller School of Medicine, Miami, FL, United States; ^6^ Department of Pathology, Molecular and Cell-based Medicine, Icahn School of Medicine at Mount Sinai, New York, NY, United States

**Keywords:** vaccine reactogenicity, infection, protective antibodies, COVID-19, SARS-CoV-2

## Abstract

**Introduction:**

The influence of pre-existing humoral immunity, inter-individual demographic factors, and vaccine-associated reactogenicity on immunogenicity following COVID vaccination remains poorly understood.

**Methods:**

Ten-fold cross-validated least absolute shrinkage and selection operator (LASSO) and linear mixed effects models were used to evaluate symptoms experienced by COVID+ participants during natural infection and following SARS-CoV-2 mRNA vaccination along with demographics as predictors for antibody (AB) responses to recombinant spike protein in a longitudinal cohort study.

**Results:**

In previously infected individuals (n=33), AB were more durable and robust following primary vaccination when compared to natural infection alone. Higher AB were associated with experiencing dyspnea during natural infection, as was the total number of symptoms reported during the COVID-19 disease course. Both local and systemic symptoms following 1^st^ and 2^nd^ dose (n=49 and 48, respectively) of SARS-CoV-2 mRNA vaccines were predictive of higher AB after vaccination. Lastly, there was a significant temporal relationship between AB and days since infection or vaccination, suggesting that vaccination in COVID+ individuals is associated with a more robust immune response.

**Discussion:**

Experiencing systemic and local symptoms post-vaccine was suggestive of higher AB, which may confer greater protection.

## 1 Introduction

The heterogeneous presentation of SARS-CoV-2 infection is associated with inter-individual factors (1, 2) including age, biological sex, comorbidities, susceptibility to the virus, exposure load, viral shedding, pre-existing binding or neutralizing antibodies (3, 4), and pre-existing cross-reactive T cells (5–7). Variability in these factors and their distinct contributions to the individual immune response has made it difficult to generalize the disease course in SARS-CoV-2-infected individuals (1, 8). Immunoassays (i.e., virus-specific serologic assays) have been used extensively throughout the COVID-19 pandemic (9). Primarily used to characterize the immune response following vaccination, assessing viability for convalescent plasma donation, and acting as a population surveillance tool (10, 11), the most pressing work remains developing correlates for protection. Neutralizing and binding titers remain well supported as protective markers (3, 12) regardless of natural infection or vaccination, including a recent study (13) which associated increased binding and neutralizing antibodies with an inverse risk for SARS-CoV-2 infection following mRNA-1273 (Moderna) vaccination.

Previous studies have evaluated change in peak post-vaccination antibody titers as a function of time (14) and the relationship between lower quantitative antibodies and disease severity (15). Additionally, evidence suggests that higher antibody titers in vaccinated, previously coronavirus disease 2019 positive (COVID+) individuals are associated with an increased degree of immune protection (16–18). Studies have also shown that vaccination with mRNA vaccines results in rapid, robust antibody production and associated reactogenicity after the first vaccine dose (19, 20) as well as following heterologous booster doses (21). Here, we investigated relationships between sociodemographic factors, reactogenicity, and immunogenicity following SARS-CoV-2 mRNA vaccination among previously SARS-CoV-2 infected (COVID+) individuals participating in our longitudinal cohort study (COVID-19 ImmuniTY study, or “CITY”). This analysis may help elucidate how underlying immunologic determinants, pre-existing immunity, and vaccine reactogenicity are associated with post-vaccination antibody titers (i.e., humoral immunogenicity) in an ethnically diverse cohort. Conclusions drawn from this study may contribute to a more personalized public health approach to future COVID-19 vaccine strategies, which could account for an individual’s demographics (e.g., age, gender, or race) or existing immunity prior to vaccination or booster receipt (22).

## 2 Methods

### 2.1 Study design and participants

#### 2.1.1 “CITY” cohort

Participants were enrolled in our Institutional Review Board (IRB) approved (#20201026), longitudinal, observational SARS-CoV-2 immunity study (n=228) known as “CITY” (COVID-19 ImmuniTY study). The study began in October 2020 at the University of Miami Miller School of Medicine, and study subjects participated in visits every 3 months for a total of 2 years from the time of enrollment. The primary objective of the CITY study is to characterize differential antibody kinetics among SARS-CoV-2 uninfected and infected individuals in a high-risk, ethnically diverse cohort. The “high-risk” designation for inclusion referred to hazardous occupational exposure (e.g., healthcare workers) but also to advanced age or other sociodemographic characteristics known to increase risk of SARS-CoV-2 morbidity and mortality. Following written informed consent, participants provided demographic details to include lifestyle habits and relevant past medical history that would preclude them to more severe SARS-CoV-2 infection-related outcomes. Those who suffered from SARS-CoV-2 infection with a documented positive nucleic acid amplification test (NAAT+) prior to entry provided evidence details regarding their past COVID-19 infection symptoms during the baseline visit to the study team.

At all regularly scheduled visits, participants prospectively answered symptom questionnaires to screen for new or recent SARS-CoV-2 infection, and blood samples were collected for serum and peripheral blood mononuclear cell (PBMC) processing. Plasma was stored at -80°C and PBMCs were cryopreserved in liquid N_2_ (23). All participants agreed to sample banking for future research use. Those who received vaccines were asked to return for two optional, additional visits at 1 week and 1 month following vaccine receipt, where they answered binary “Yes/No” questions in a survey about their symptoms following vaccination. Vaccine-related symptoms were rated on a Likert scale, where a “0” indicated no symptoms and a “10” indicated the highest symptom severity. Blood samples were collected and processed as described above.

#### 2.1.2 CITY sub-cohort analysis (COVID+ unvaccinated and COVID+ vaccinated participants)

For this study, we included three groups, comprising of CITY participants enrolled between October 2020 – June 2021 with a history of COVID-19 prior to vaccination (i.e., natural infection (NI)), COVID-19+ participants who received Dose 1 (NI + dose 1 (D1)) of an mRNA vaccine, and fully vaccinated COVID-19+ participants who received Dose 1 and Dose 2 (NI + dose 2 (D2); deemed “fully vaccinated”) of an mRNA vaccine. Pfizer (BNT162b2) and Moderna (mRNA-1273) were the primary options available (9) during the enrollment period; thus, participants who only received one dose or received Johnson & Johnson (n=4 across the entire cohort) were excluded from this analysis. Additionally, participants who were administered their second dose of an mRNA vaccine >7 days after or <4 days before the recommended (24) number of days after the first dose (21 days for Pfizer; 28 days for Moderna) were excluded as well in order to account for the temporal, transient nature of post-vaccine reactogenicity and the subsequent immune response to best reflect the general population. Individuals with suspected (>2-fold increase in Ab) or confirmed (with a NAAT+ test) reinfection or breakthrough infection were also excluded. Individuals infected with SARS-CoV-2 after June 2021 were also excluded in order to control for changes in variant-specific antigenic profiles and related changes in disease presentation (i.e., the Delta VOC). Further, limited symptomatic primary infections occurred after this June 2021 as most individuals were vaccinated during this period.

In total, 32 participants overlap between the naturally infected, pre-vaccine (NI) and post-vaccination (NI + D1; NI + D2) groups. Among those who met criteria for inclusion in the sub-analysis, all samples provided during the baseline visit and thereafter were included. Of note, participants were considered “fully vaccinated” 14 days after they received Dose 2.

### 2.2 Enzyme-linked immunosorbent assay

SARS-CoV-2 ELISAs were performed using a well-described assay developed by the Icahn School of Medicine at Mount Sinai (10, 11). Discrete titers were reported in values of 1:100, 1:200, 1:400, 1:800, 1:1600, 1:3200, 1:6400, 1:12800, 1:25600, 1:51200, 1:102400, and 1:204800. The limit of detection was set at 1:100.

### 2.3 Statistical analysis

SARS-CoV-2 antibody titers were log_2_-transformed before all statistical analyses. To model the bi-phasic change in antibodies over time, we utilized a generalized additive model (GAM) incorporating the rate of both antibody decay following natural infection (following COVID+ participants’ last positive SARS-CoV-2 test [LPT] result) and full vaccination (≥14 days after the second mRNA vaccine dose) [24]. The GAM modelled antibody titers with a smoothed function for number of days elapsed using a cubic regression with 3 knots as well as the fixed effect of vaccination status (NI and NI + D2). We then replicated the above as a linear mixed-effects model (LMM), where we incorporated the same fixed effects but included participants as a random-intercepts effect to control for individual differences. The rates of log-transformed antibody titer decay along with the limit of detection of our assay were used to estimate the number of days that the antibodies remain detectable after both natural infection and full vaccination.

Ten-fold cross-validated least absolute shrinkage and selection operator (LASSO) models were employed as a feature-selection and regularization technique. LASSO models were tuned to select the simplest model within one standard error of the lowest value root-mean-square error accuracy metric that included at least two predictors (**Supplementary Table 1**). Four models with identical demographic variables were constructed while controlling for time or days since LPT, 1st dose, or 2nd dose, respectively, including: 1) the effect of infection symptoms on the antibody response post-infection, 2) the effect of infection symptoms on the antibody response post full vaccination, 3) the effect of dose 1 vaccine symptoms on the antibody response post full vaccination, and 4) the effect of dose 2 vaccine symptoms on the antibody response post full vaccination. The selected predictors from each of the best-fitting cross-validated LASSO models were then included as fixed effects in follow-up LMMs with by-participant random intercepts, allowing us to control for individual differences. For significant categorical fixed effects from the LMMs, we conducted *post-hoc* Tukey tests to confirm directionality and to correct for multiple comparisons.

Additional linear regressions were used to investigate effects of each symptom following natural infection alone (NI), natural infection and the 1^st^ dose of SARS-CoV-2 mRNA vaccination (NI + D1), and natural infection and the 2^nd^ dose of vaccination (NI + D2) and explore possible relationships between demographics factors on peak antibody titer levels following full vaccination. Analyses were performed using R statistical software Version 4.1.1 (25). Generalized additive modeling, cross-validation, LASSO modelling, LMM, and *post-hoc* Tukey tests were conducted with the R packages *mgcv* (26), *caret* (27), *glmnet* (28)*, nlme* (29), and *glht* (30), respectively, while the linear modelling, Mann-Whitney U tests, and Kruskal-Wallace tests were performed using the R package *stats* (25). Plots were produced using the *ggplot2* (31).

## 3 Results

### 3.1 Characteristics of the study population

Demographic characteristics are detailed in **Table 1**. Thirty-three participants with a history of COVID-19 were included in our post infection cohort (NI [natural infection alone]). Following natural infection (prior to mRNA vaccine receipt), the median number of days since last PCR-positive SARS-CoV-2 test to the baseline study visit was 101 days. For the post dose 1 (NI + D1 [natural infection and primary vaccine dose 1]) and post dose 2 (NI + D2 [natural infection and primary vaccine dose 2]) analysis, we included 49 and 48 participants, respectively. Median days from LPT to Dose 1 and Dose 2 were 99 and 127 days, respectively. No participants were known to be immunocompromised. All infections were deemed to be mild, with none requiring hospitalization.

**Table 1:** Characteristics of the study population following natural infection, 1st dose of vaccine, and 2nd dose of vaccine.

	Natural Infection (NI)	Natural Infection + Vaccine Dose 1 (NI + D1)	Natural Infection + Vaccine Dose 2 (NI + D2)
**n**	33	49	48
**Gender, M/F**	14/19	17/32	16/32
**Race**	White [25 (76%)] Black/African American [2 (6%)] Asian [1 (3%)] Other [5 (15%)]	White [39 (80%)] Black/African American [2 (4%)] Asian [2 (4%)] Other [6 (12%)]	White [(39 (81%)] Black/African American [2 (4%)] Asian [2 (4%)] Other [5 (110%)]
**Ethnicity, Hispanic/Not Hispanic**	14/19 (42%/58%)	22/27 (45%/55%)	21/27 (44%/56%)
**Median age [Range]**	39 [20-76]	39 [20-78]	39 [20-78]
**Vaccine manufacturer, Pfizer/Moderna**	–	31/18 (63%/37%)	30/18 (62.5%/37.5%)
**Median days since COVID diagnosis by PCR to entry SD Range**	101 74.87 8 - 292	–	–
**Median days since COVID diagnosis by PCR to vaccination 95% CI**	–	99 72 – 159	127 102-180

### 3.2 Symptoms reported following infection and vaccination

The highest reported symptom during the vaccine-naïve COVID-19 course was fatigue (63%) (**Table 2**). Other highly reported symptoms included anosmia (55%), congestion (53%), and myalgias/muscle aches (57%). Following dose 1, the most common symptoms were injection site pain (51%), headache (29%), and fatigue (29%). Similarly, the most common symptoms reported following the second mRNA vaccination (dose 2) were injection site pain (53%), fatigue (39%), and myalgias (29%) (**Table 2**).

**Table 2:** Symptoms experienced by the study cohort following natural infection, 1st dose of vaccine, and 2nd dose of vaccine.

	Natural Infection (NI)^b^	Natural Infection + Vaccine Dose 1 (NI + D1)	Natural Infection + Vaccine Dose 2 (NI + D2)
Symptoms Reported During SARS-CoV-2 Infection^a^			
Asymptomatic	10 (20%)	–	–
Anosmia	27 (55%)	–	–
Congestion/rhinorrhea	26 (53%)	–	–
Cough	23 (47%)	–	–
Difficulty breathing	12 (24%)	–	–
Dysgeusia	26 (53%)	–	–
Fatigue	31 (63%)	–	–
Fever	18 (37%)	–	–
Myalgias	28 (57%)	–	–
Nausea or vomiting	5 (10%)	–	–
Sore Throat	15 (31%)	–	–
Upset Stomach	14 (29%)	–	–
Local Symptoms Reported Following Vaccination^a^			
Injection site pain	–	25 (51%)	26 (53%)
Injection site redness	–	2 (4%)	4 (8%)
Injection site swelling	–	4 (8%)	5 (10%)
Systemic Symptoms Reported Following Vaccination^a^			
Asymptomatic	–	22 (45%)	20 (41%)
Chills	–	9 (18%)	11 (22%)
Fatigue	–	14 (29%)	19 (39%)
Fever	–	10 (20%)	10 (20%)
Headache	–	14 (29%)	12 (24%)
Myalgias/Muscle Aches	–	12 (24%)	14 (29%)

Each count is the number of individuals who self-reported each symptom at the timepoint listed in each column. Percentages are based on the total n for each column.

a All symptoms reported during SARS-CoV-2 Infection, as well as local and systemic symptoms following vaccination were included in LASSO modeling as shown in **Supplementary Table 1**.

b All samples collected following natural infection were included for analysis.

### 3.3 In previously infected individuals, antibody titers are more robust following full-vaccination as compared to post natural infection

**Figure 1:**
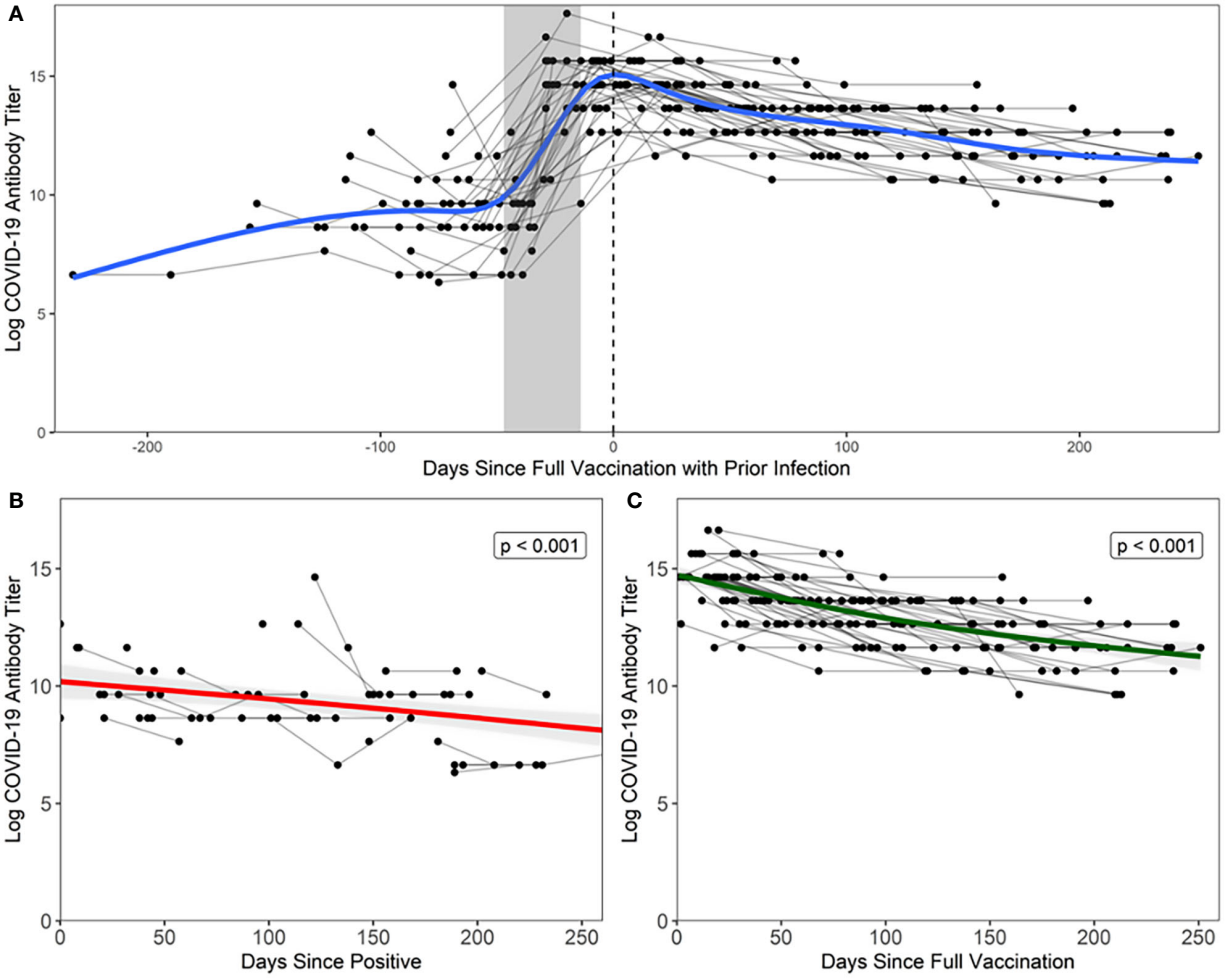
Antibody response following natural infection and vaccination Each black point represents a sample from a participant, grey lines connect points from the same participant, and the grey shaded area represents the maximum number of days between doses relative to date of full vaccination (14 days after second dose, regardless of vaccine manufacturer). **(A)** Days since full vaccination *vs*. log titers over time. t=0 on the x-axis represents the day when COVID+ participants became fully vaccinated (2 weeks after second vaccination). Bi-phasic, generalized additive model (GAM) is visualized by a blue line. **(B)** In unvaccinated COVID+ participants, log_2_ antibody titers decay at a rate of -0.010 per day after last positive COVID-19 test result. Fitted linear model is visualized by a red line. Note that three points were excluded from the above figure due to the temporal scale used to graphically depict the data but are included in the analyses herein. **(C)** In vaccinated COVID+ participants, log2 antibody titers decay at a rate of -0.015 per day after full vaccination. Fitted linear model is visualized by a green line.

There was a more robust antibody response immediately following full vaccination (**Figure 1C**) when compared to the antibody response following natural infection (estimate = 4.117, *t* = 12.950, *p* = < 0.001) (**Figure 1B**), where peak log_2_ antibody titers were greater in the vaccination with prior infection (14.517) than in natural infection (9.217). This is illustrated in **Figure 1A**, where natural infection and post-full vaccination titers were included in a bi-phasic model to show longitudinal antibody responses. Infected individuals had a slower rate of antibody titer decay (-0.010 *vs* -0.015 log per day), though this effect was small (estimate = -0.005, *t* = 2.351, *p* = 0.020).

LMMs confirmed our findings that antibody titer declined faster following full vaccination than in natural infection (estimate = -0.006, *F* =11.238, *p <*0.001). Of note, the combination of natural infection followed by vaccination, or so-called “hybrid immunity”, elicits a more durable antibody response than natural infection alone (estimate = 4.138, *F* = 794.623, *p* < 0.0001), as log_2_ antibody titers were predicted to remain detectable for a longer period of time following natural infection and full vaccination (550 days) than natural infection alone (464 days).

### 3.4 Days elapsed and symptoms reported during infection influence antibody titers

As shown in **Table 3**, difficulty breathing during infection (estimate = 1.590, *F* = 5.684, *p* = 0.024) and days elapsed since LPT (estimate = -0.006, F = 9.912, *p* = .004) were significant main effects in predicting antibody titers following natural infection. *Post-hoc* testing confirmed that antibody titers were elevated in individuals who experienced difficulty breathing (*z* = 2.612; *p* = 0.009).

**Table 3:** Symptoms and demographic factors influence antibody responses following natural infection and full vaccination.

Category	Predictor	Coefficient^a^	F-value^a^	p-value^a^	Pr(>|z|)^b^
Demographics, symptoms reported during infection and titers following natural infection					
*Days elapsed*	Days since positive test	-0.01	9.991	0.004*	–
*Symptoms reported during infection*	Difficulty breathing	0.58	5.684	0.024*	0.020*
	Subjective symptom severity	0.00	2.524	0.123	**–**
Demographics, symptoms reported during infection and titers following full vaccination					
*Days elapsed*	Days since full vaccination	-0.01	258.176	<0.0001*	–
*Symptoms reported during infection*	Anosmia during infection	0.31	2.910	0.096	–
*Demographics*	Age	0.01	6.000	0.019*	–
	Ethnicity (Hispanic)	0.20	5.265	0.027*	0.062
	Flu vaccinated	-0.37	3.612	0.064	–
	Gender (Male)	-0.09	2.402	0.129	–
	Vaccine manufacturer (Pfizer)	-0.16	0.306	0.583	–
Demographics, symptoms reported following Dose 1, and titers following full vaccination					
*Days elapsed*	Days since full vaccination	-0.01	262.855	<0.0001*	–
*Symptoms reported after 1^st^ vaccine dose*	Chills after 1^st^ dose	0.34	4.915	0.032*	0.085
	Injection site redness after 1^st^ dose	0.91	4.330	0.044*	0.038*
*Demographics*	Age	0.01	6.960	0.012*	–
	Ethnicity (Hispanic)	0.28	4.583	0.038*	0.017*
	Flu vaccinated	-0.15	1.771	0.191	–
	Gender (Male)	-0.08	2.288	0.138	–
Demographics, symptoms reported following Dose 2, and titers following full vaccination					
*Days elapsed*	Days since full vaccination	-0.01	259.745	<0.0001*	–
*Symptoms reported after 2^nd^ dose*	Fever	0.61	11.154	0.002*	0.003*
	Injection site swelling after 2^nd^ dose	0.31	1.579	0.216	–
	Subjective symptom severity	-0.03	3.064	0.088	–
*Demographics*	Age	0.01	7.652	0.009*	–
	Ethnicity (Hispanic)	0.32	6.683	0.013*	0.006*
	Flu vaccinated	-0.29	4.405	0.042*	0.166
	Gender (Male)	-0.05	3.452	0.071	–

Four LASSO models and linear mixed-effects (LME) p-values were generated to assess the predictive value of categorical and discrete variables while controlling for between-subjects’ differences. Significant categorical LASSO-generated variables underwent additional post-hoc Tukey to test for directionality and to correct for multiple comparisons. A p-value <0.05 was considered statistically significant.

a LME for predictors of main effects.

b Post-hoc Tukey test for directionality of categorical variables due to significant main effects.

When modeling demographics and symptoms at infection to predict the antibody response after full vaccination, we found that days elapsed since full vaccination (estimate = -0.014; *F* = 258.176; *p* < 0.0001), increased age (estimate = 0.018; *F* = 6.000; *p* = 0.019), and ethnicity (Hispanic) (estimate = 0.456; *F* = 5.265; *p* = 0.018) were significant main effects, though no categorical variables were significant after *post-hoc* testing.

### 3.5 Symptoms following vaccination are predictive of higher antibody titers after full vaccination

Local and systemic symptoms following the 1^st^ dose of SARS-CoV-2 mRNA vaccines were predictive of higher antibody titers after full vaccination. As seen in **Table 3**, the results of the LMM show that days elapsed since full vaccination (estimate = -0.014, *F* = 262.855, *p* < 0.0001), chills (estimate = 0.541; *F* = 4.915; *p* = 0.032), injection site redness (estimate = 1.243; *F* = 4.330; p = 0.044), age (estimate = 0.021; *F* = 6.960; *p* = 0.012), and ethnicity (estimate = 0.562, *F* = 4.583, *p* = 0.038) were significant. Following *post-hoc* Tukey testing on the significant categorical main effects, we found that injection site redness (*z* = 2.081, *p* = 0.038) and ethnicity (Hispanic) (*z* = 2.382, *p* = 0.017) were significant, while the main effect of chills was not.

We also examined demographics and symptoms reported during the 2^nd^ dose of SARS-CoV-2 mRNA vaccination and their effect on the antibody response after full vaccination. Days since full vaccination (estimate = -0.014; *F* = 259.745; *p* < 0.0001), age (estimate = 0.023; *F* = 7.652; *p* = 0.009), identifying as Hispanic (estimate = 0.609; *F* = 6.683; *p* = 0.013), fever (estimate = 0.839; *F* = 11.154, *p* = 0.002), and influenza vaccination (estimate = -0.475; *F* = 4.405; *p* = 0.042) were observed to be significant. Fever and ethnicity (Hispanic) were found to be statistically significant (*z* = 3.016, *p* = 0.003; *z* = 2.735, *p* = 0.006, respectively) following *post-hoc* testing, though influenza vaccination was not.

### 3.6 The number of symptoms observed during infection are associated with higher peak antibody titers post full-vaccination

The number of symptoms self-reported during infection significantly predicted peak antibody titers after full vaccination (estimate = 0.10, *t* = 2.10, Pearson’s *r* = 0.296; *p* = .041). Additional linear models were conducted for the number of symptoms reported as a function of demographics, where we found that the number of symptoms self-reported during infection was significantly influenced by self-identifying as White (estimate = 4.679, *t* = 2.153, *p* = 0.037). No other demographic factors was significant.

## 4 Discussion

The goal of this study was to investigate the role of demographics, pre-existing immunity, and symptomatology following infection and vaccination to ascertain whether they independently or collectively are associated with immunogenicity following mRNA vaccination against SARS-CoV-2 infection in individuals previously infected with SARS-CoV-2. Our results demonstrate higher durability and robustness of antibody titers despite a faster rate of antibody decay following vaccination, which supports previously reported findings (32) for SARS-CoV-2 mRNA vaccines. Unsurprisingly, our results also demonstrate that a larger temporal gap between an individual’s LPT and antigen testing predicts decline of antibody titers over time. We also found that in previously infected individuals, SARS-CoV-2 mRNA vaccines result in a more robust antibody response than that following infection alone.

Following infection alone, the number of symptoms reported and difficulty breathing during the COVID-19 course were predictive of higher antibody titers. This result supports existing evidence (33, 34) that individuals who report a more severe or symptomatic SARS-CoV-2 infection have higher peak titers than asymptomatic individuals. After receiving dose 1, injection site redness was found to be significantly predictive for higher antibody titers following full vaccination. Interestingly, after dose 2 we found that fever was significantly predictive for higher antibody titers following full vaccination. It should be noted that asymptomatic individuals mounted robust immune responses as well.

In previously infected individuals, SARS-CoV-2 mRNA vaccines result in a more robust antibody response than that following infection alone. Indeed, in individuals with “hybrid immunity”, antibody titers following full vaccination peak at 4-fold higher than those following naturally acquired immunity and appear to persist at detectable levels for >500 days following vaccination. One explanation for this increased response could be the presence of pre-existing memory T and B cell responses developed during natural infection. These cells might enhance a secondary immune response following vaccination similar to that of a booster immunization. In addition to bolstering the current CDC recommendations (35) that individuals previously infected with SARS-CoV-2 receive vaccination, our results provide additional, longitudinal support for this measure.

Intrinsic factors, such as age and gender are thought to influence immunogenicity (36). Though our data support higher peak antibody titers following infection and vaccination with increasing age (33, 37) it should be noted that the median age across all groups included in this analysis was 39 years with no participant above 80 years of age and therefore should not be interpreted as a superior post-infection or post-vaccine antibody responses in the elderly. We expect that old age will be associated with poorer vaccine responses as has been described previously (38) but this was not evaluated here. Notably, our study was conducted in Miami-Dade County, an international, multi-cultural hub with a largely Hispanic and bilingual population. Our analysis demonstrated a significant relationship between Hispanic ethnicity and higher antibody titers over time at nearly every time point of interest, including infection where the analysis approached significance (*p* = 0.0624). Other groups have demonstrated higher rates of Hispanic SARS-CoV-2 seroconversion when compared to other ethnicities (39, 40) and have found that Hispanic ethnicity is linked to higher rates of seroprotection and seroconversion following H1N1 monovalent vaccination (41, 42), but future studies with a large number of participants are needed to support a generalizable trend for antibody magnitude over time in this population. Related, we also found that influenza vaccination was associated with higher antibody titers, though it was not significant following *post-hoc* testing. Though the biological relevance of this finding is unknown, we previously showed that specific CD4 responses to influenza A(H1N1) correlate with SARS-CoV-2 specific CD4 T-cells, suggesting a protective effect of pre-existing influenza specific T-cells (7). We speculate that this provides evidence of healthy and “trained” immune systems within our cohort, wherein epigenetic and metabolic reprogramming have augmented innate immune cells that enhance adaptive immunity to increase SARS-CoV-2 specific responses (7, 43).

Our study has several limitations, namely that sample sizes for each cohort examined were small due to variability in vaccination timelines and participant scheduling. Some individuals were excluded due to a confounding effect on our predictive modeling, which is controlled for by the fixed effect of time relative to desired endpoints (i.e.: infection and vaccination). The natural infection group was further limited by the study timeline, as the first SARS-CoV-2 vaccination became available shortly after enrollment began and therefore limited the number of naturally infected individuals, we were able to follow longitudinally. Additionally, our analysis only included quantitative binding antibody titers. Though recent work has demonstrated that higher binding antibodies correlate to higher neutralizing antibodies (13), expansive, multi-center longitudinal studies profiling the cellular and humoral response are needed. Comprehensive future work would benefit from characterizing immunogenetic determinants for SARS-CoV-2 vaccine effectiveness (44, 45) *via* a so-called “Adversomics” approach (46) and should include profiling of quantitative binding antibodies, neutralizing and non-neutralizing antibodies, memory B-cells, and T-cell responses, as immune protection appears to be contingent on all three tiers of the immune response (47, 48).

Finally, some of the predictors used in our statistical analysis were found to be significant in one test but not in *post-hoc* tests. Large, longitudinal studies are required to confirm a significant group difference, but the predictors utilized herein should be included in future analyses. Our bivariate analysis of symptoms experienced following the 1^st^ and 2^nd^ doses failed to demonstrate that individual symptoms can influence peak antibody titers following full vaccination. The same was true for race and ethnicity, which were not found to be significantly predictive for peak titers, though we contend that this is because these models failed to control for individual differences, or intercepts, to account for between-subjects’ variability.

In conclusion, this study demonstrates that a combination of systemic and local symptoms is predictive of higher antibody titers, which may correlate to a higher degree of protection. Additional studies are needed to understand the role of immunologic determinants (including underlying genetic polymorphisms that influence immune cell activation/differentiation, etc.) for protection against SARS-CoV-2 infection and breakthrough infection in the age of boosters and variants capable of immune escape, as symptom profiles seem to be variant-specific (49, 50). Repeating this type of analysis at the population level along with fully characterized adaptive cellular responses will be critical in providing personalized recommendations for future vaccine measures, including recommendations for optimal booster timing.

## Data availability statement

The raw data supporting the conclusions of this article will be made available by the authors, without undue reservation.

## Ethics statement

The studies involving human participants were reviewed and approved by University of Miami Institutional Review Board. The patients/participants provided their written informed consent to participate in this study.

## Author contributions

SaP conceived the Miami cohort study and EW, AK, SuP contributed to conception and design of the manuscript. VS, FT, DM organized the database. AK performed the statistical analysis and generated data visualizations. EW, VS, AN, DM wrote sections of the manuscript. EW, AN, DM, FT, JMC, DB, and GS provided technical assistance or collected data for this manuscript. MH, FK, JMC, SaP, and SuP provided oversight of the study and edited the manuscript. All authors contributed to manuscript revision, read, and approved the submitted version.

## References

[1] Rodebaugh TL Frumkin MR Reiersen AM Lenze EJ Avidan MS Miller JP. Acute symptoms of mild to moderate COVID-19 are highly heterogeneous across individuals and over time. Open Forum Infect Dis (2021) 8(3):ofab090. doi: 10.1093/ofid/ofab090 33796601 PMC7989225

[2] Zimmermann P Curtis N. Factors that influence the immune response to vaccination. Clin Microbiol Rev (2019) 32(2):e00084–18. doi: 10.1128/CMR.00084-18 PMC6431125 30867162

[3] Khoury DS Cromer D Reynaldi A Schlub TE Wheatley AK Juno JA. Neutralizing antibody levels are highly predictive of immune protection from symptomatic SARS-CoV-2 infection. Nat Med (2021) 27(7):1205–11. doi: 10.1038/s41591-021-01377-8 34002089

[4] Garcia-Beltran WF Lam EC Astudillo MG Yang D Miller TE Feldman J. COVID-19-neutralizing antibodies predict disease severity and survival. Cell (2021) 184(2):476–88.e11. doi: 10.1016/j.cell.2020.12.015 33412089 PMC7837114

[5] da Silva Antunes R Pallikkuth S Williams E Dawen Yu E Mateus J Quiambao L. Differential T-cell reactivity to endemic coronaviruses and SARS-CoV-2 in community and health care workers. J Infect Dis (2021) 224(1):70–80. doi: 10.1093/infdis/jiab176 33822097 PMC8083569

[6] Mateus J Grifoni A Tarke A Sidney J Ramirez SI Dan JM. Selective and cross-reactive SARS-CoV-2 T cell epitopes in unexposed humans. Science (2020) 370(6512):89–94. doi: 10.1126/science.abd3871 32753554 PMC7574914

[7] Pallikkuth S Williams E Pahwa R Hoffer M Pahwa S. Association of flu specific and SARS-CoV-2 specific CD4 T cell responses in SARS-CoV-2 infected asymptomatic heath care workers. Vaccine (2021) 39:6019–24. doi: 10.1016/j.vaccine.2021.08.092 PMC8403669 34531078

[8] Thevarajan I Buising KL Cowie BC. Clinical presentation and management of COVID-19. Med J Aust (2020) 213(3):134–9. doi: 10.5694/mja2.50698 PMC7404664 32677734

[9] FDA. Coronavirus disease 2019 (COVID-19). EUA Informatin (2022). Available at: https://www.fda.gov/emergency-preparedness-and-response/mcm-legal-regulatory-and-policy-framework/emergency-use-authorizationvaccines.

[10] Amanat F Stadlbauer D Strohmeier S Nguyen THO Chromikova V McMahon M. A serological assay to detect SARS-CoV-2 seroconversion in humans. Nat Med (2020) 26(7):1033–6. doi: 10.1038/s41591-020-0913-5 PMC8183627 32398876

[11] Stadlbauer D Amanat F Chromikova V Jiang K Strohmeier S Arunkumar GA. SARS-CoV-2 seroconversion in humans: A detailed protocol for a serological assay, antigen production, and test setup. Curr Protoc Microbiol (2020) 57(1):e100. doi: 10.1002/cpmc.100 32302069 PMC7235504

[12] Feng S Phillips DJ White T Sayal H Aley PK Bibi S. Correlates of protection against symptomatic and asymptomatic SARS-CoV-2 infection. Nat Med (2021) 27(11):2032–40. doi: 10.1038/s41591-021-01540-1 PMC8604724 34588689

[13] Gilbert PB Montefiori DC McDermott AB Fong Y Benkeser D Deng W. Immune correlates analysis of the mRNA-1273 COVID-19 vaccine efficacy clinical trial. Science (2022) 375(6576):43–50. doi: 10.1126/science.abm3425 PMC9017870 34812653

[14] Crawford KHD Dingens AS Eguia R Wolf CR Wilcox N Logue JK. Dynamics of neutralizing antibody titers in the months after severe acute respiratory syndrome coronavirus 2 infection. J Infect Dis (2021) 223(2):197–205. doi: 10.1093/infdis/jiaa618 33535236 PMC7543487

[15] Nagura-Ikeda M Imai K Kubota K Noguchi S Kitagawa Y Matsuoka M. Clinical characteristics and antibody response to SARS-CoV-2 spike 1 protein using VITROS anti-SARS-CoV-2 antibody tests in COVID-19 patients in Japan. J Med Microbiol (2021) 70(4):001291. doi: 10.1099/jmm.0.001291 33861191 PMC8289209

[16] Callegaro A Borleri D Farina C Napolitano G Valenti D Rizzi M. Antibody response to SARS-CoV-2 vaccination is extremely vivacious in subjects with previous SARS-CoV-2 infection. J Med Virol (2021) 93(7):4612–5. doi: 10.1002/jmv.26982 PMC8250392 33788281

[17] Lozano-Ojalvo D Camara C Lopez-Granados E Nozal P Del Pino-Molina L Bravo-Gallego LY. Differential effects of the second SARS-CoV-2 mRNA vaccine dose on T cell immunity in naive and COVID-19 recovered individuals. Cell Rep (2021) 36(8):109570. doi: 10.1016/j.celrep.2021.109570 34390647 PMC8332924

[18] Mungmunpuntipantip R Wiwanitkit V. Antibody response to SARS-CoV-2 vaccination, previous SARS-CoV-2 infection, and change to single-dose vaccination. J Med Virol (2021) 93(12):6474. doi: 10.1002/jmv.27263 34374991 PMC8426949

[19] Kelsen SG Braverman AS Aksoy MO Hayman JA Patel PS Rajput C. SARS-CoV-2 BNT162b2 vaccine–induced humoral response and reactogenicity in individuals with prior COVID-19 disease. JCI Insight (2022) 7(4). doi: 10.1172/jci.insight.155889 PMC8876462 35019861

[20] Ontañón J Blas J de Cabo C Santos C Ruiz-Escribano E García A. Influence of past infection with SARS-CoV-2 on the response to the BNT162b2 mRNA vaccine in health care workers: Kinetics and durability of the humoral immune response. EBioMedicine (2021) 73:103656. doi: 10.1016/j.ebiom.2021.103656 34740112 PMC8556513

[21] Pereson MJ Amaya L Neukam K Baré P Echegoyen N Badano MN. Heterologous gam-COVID-vac (sputnik V)/mRNA-1273 (moderna) vaccination induces a stronger humoral response than homologous sputnik V in a real-world data analysis. Clin Microbiol Infection (2022) 28(10):1382–8. doi: 10.1016/j.cmi.2022.05.009 PMC9112602 35595128

[22] Tsang JS Dobaño C VanDamme P Moncunill G Marchant A Othman RB. Improving vaccine-induced immunity: Can baseline predict outcome? Trends Immunol (2020) 41(6):457–65. doi: 10.1016/j.it.2020.04.001 PMC7142696 32340868

[23] HANC. Cross-network PBMC processing standard operating procedure. HIV/AIDS Network Coordination (2018). pp. 1–45. Available at: https://www.hanc.info/content/dam/hanc/documents/laboratory/cross-network-procedures-sops/HANC-LAB-P0001_v6.0_2018-04-26_PBMC_SOP.pdf

[24] CDC. Interim clinical considerations for use of COVID-19 vaccines currently approved or authorized in the united states. Centers for Disease Control and Prevention (2022). Available at: https://www.cdc.gov/vaccines/covid-19/clinical-considerations/covid-19-vaccines-us.html

[25] R Development Core Team. R: A language and environment for statistical computing. R Core Team (2021). Availabe at: https://www.R-project.org

[26] Wood SN. Stable and efficient multiple smoothing parameter estimation for generalized additive models. J Am Stat Assoc (2022) 99:673–86. doi: 10.1198/016214504000000980 https://cran.r-project.org/web/packages/nlme/nlme.pdf

[27] Kuhn M. Caret: Classification and regression training. Springer: Verlag New York (2021). Availabe at: https://github.com/topepo/caret/

[28] Friedman J Hastie T Tibshirani R. Regularization paths for generalized linear models *via* coordinate descent. J Stat Software (2010) 33(1):1–22. doi: 10.18637/jss.v033.i01 PMC2929880 20808728

[29] Pinheiro J Bates D DebRoy S Sarkar D R Core Team. Nlme: Linear and nonlear mixed effects models. R package version 31-1522021. (2022). Available at: https://cran.r-project.org/web/packages/nlme/nlme.pdf

[30] Hothorn T Bretz F Westfall P. Simultaneous inference in general parametric models. Biometric J (2008) 50(3):346–63. doi: 10.1002/bimj.200810425 18481363

[31] Wickham H. ggplot2: Elegant graphics for data analysis. New York: Springer-Verlag (2016).

[32] Levin EG Lustig Y Cohen C Fluss R Indenbaum V Amit S. Waning immune humoral response to BNT162b2 covid-19 vaccine over 6 months. New Engl J Med (2021) 385(24):e84. doi: 10.1056/NEJMoa2114583 34614326 PMC8522797

[33] Röltgen K Powell AE Wirz OF Stevens BA Hogan CA Najeeb J. Defining the features and duration of antibody responses to SARS-CoV-2 infection associated with disease severity and outcome. Sci Immunol (2020) 5(54). doi: 10.1126/sciimmunol.abe0240 PMC7857392 33288645

[34] Gudbjartsson DF Norddahl GL Melsted P Gunnarsdottir K Holm H Eythorsson E. Humoral immune response to SARS-CoV-2 in Iceland. New Engl J Med (2020) 383(18):1724–34. doi: 10.1056/NEJMoa2026116 PMC7494247 32871063

[35] CDC. Science brief: SARS-CoV-2 infection-induced and vaccine-induced immunity. (2021). 34748301

[36] Hervé C Laupèze B Del Giudice G Didierlaurent AM Tavares Da Silva F. The how’s and what’s of vaccine reactogenicity. NPJ Vaccines (2019) 4(1):39. doi: 10.1038/s41541-019-0132-6 31583123 PMC6760227

[37] Dan JM Mateus J Kato Y Hastie KM Yu ED Faliti CE. Immunological memory to SARS-CoV-2 assessed for up to 8 months after infection. Science (2021) 371(6529). doi: 10.1126/science.abf4063 PMC7919858 33408181

[38] Müller L Andrée M Moskorz W Drexler I Walotka L Grothmann R. Age-dependent immune response to the Biontech/Pfizer BNT162b2 coronavirus disease 2019 vaccination. Clin Infect Dis (2021) 73(11):2065–72. doi: 10.1093/cid/ciab381 PMC8135422 33906236

[39] Kennedy JL Forrest JC Young SG Amick B Williams M James L. Temporal variations in seroprevalence of SARS-CoV-2 infections by race and ethnicity in Arkansas. medRxiv (2021). doi: 10.1101/2021.07.15.21260213 PMC9045955 35493126

[40] Rogawski McQuade ET Guertin KA Becker L Operario D Gratz J Guan D. Assessment of seroprevalence of SARS-CoV-2 and risk factors associated with COVID-19 infection among outpatients in Virginia. JAMA Network Open (2021) 4(2):e2035234–e. doi: 10.1001/jamanetworkopen.2020.35234 PMC7871191 33555331

[41] Pass RF Nachman S Flynn PM Muresan P Fenton T Cunningham CK. Immunogenicity of licensed influenza a (H1N1) 2009 monovalent vaccines in HIV-infected children and youth. J Pediatr Infect Dis Soc (2013) 2(4):352–60. doi: 10.1093/jpids/pit040 PMC3869470 24363932

[42] Arguedas A Soley C Lindert K. Responses to 2009 H1N1 vaccine in children 3 to 17 years of age. New Engl J Med (2010) 362(4):370–2. doi: 10.1056/NEJMc0909988 20042746

[43] Netea MG Domínguez-Andrés J Barreiro LB Chavakis T Divangahi M Fuchs E. Defining trained immunity and its role in health and disease. Nat Rev Immunol (2020) 20(6):375–88. doi: 10.1038/s41577-020-0285-6 PMC7186935 32132681

[44] Smatti MK Alkhatib HA Al Thani AA Yassine HM. Will host genetics affect the response to SARS-CoV-2 vaccines? historical precedents. Front Med (2022) 9. doi: 10.3389/fmed.2022.802312 PMC8962369 35360730

[45] Valdés-Fernández BN Duconge J Espino AM Ruaño G. Personalized health and the coronavirus vaccines-do individual genetics matter? Bioessays (2021) 43(9):e2100087. doi: 10.1002/bies.202100087 34309055 PMC8390434

[46] Whitaker JA Ovsyannikova IG Poland GA. Adversomics: A new paradigm for vaccine safety and design. Expert Rev Vaccines (2015) 14(7):935–47. doi: 10.1586/14760584.2015.1038249 PMC4630804 25937189

[47] Rydyznski Moderbacher C Ramirez SI Dan JM Grifoni A Hastie KM Weiskopf D. Antigen-specific adaptive immunity to SARS-CoV-2 in acute COVID-19 and associations with age and disease severity. Cell (2020) 183(4):996–1012.e19. doi: 10.1016/j.cell.2020.09.038 33010815 PMC7494270

[48] Vardhana S Baldo L Morice WG 2nd Wherry EJ. Understanding T cell responses to COVID-19 is essential for informing public health strategies. Sci Immunol (2022) 7(71):eabo1303. doi: 10.1126/sciimmunol.abo1303 35324269 PMC10344642

[49] Graham MS Sudre CH May A Antonelli M Murray B Varsavsky T. Changes in symptomatology, reinfection, and transmissibility associated with the SARS-CoV-2 variant B.1.1.7: An ecological study. Lancet Public Health (2021) 6(5):e335–e45. doi: 10.1016/S2468-2667(21)00055-4 PMC8041365 33857453

[50] Whitaker M Elliott J Bodinier B Barclay W Ward H Cooke G. Variant-specific symptoms of COVID-19 in a study of 1,542,510 adults in England. Nat Commun (2022) 13(1):6856. doi: 10.1038/s41467-022-34244-2 36369151 PMC9651890

[51] Williams EC Kizhner A Stark VS Nawab A Muniz DD Tribin FE. Predictors for reactogenicity and humoral immunity to SARS-CoV-2 following infection and mRNA vaccination: A regularized mixed-effects modelling approach. medRxiv (2022). doi: 10.1101/2022.04.05.22273450 PMC9949966 36845120

